# Colorectal cancer is associated with increased circulating lipopolysaccharide, inflammation and hypercoagulability

**DOI:** 10.1038/s41598-020-65324-2

**Published:** 2020-05-29

**Authors:** Greta M. de Waal, Willem J. S. de Villiers, Timothy Forgan, Timothy Roberts, Etheresia Pretorius

**Affiliations:** 1 Department of Physiological Sciences, Stellenbosch University, Stellenbosch, Private Bag X1 Matieland, 7602 South Africa; 2 Department of Internal Medicine, Stellenbosch University, Stellenbosch, Private Bag X1 Matieland, 7602 South Africa; 3 Consultant Colorectal Surgeon, Division of Surgery, Faculty of Medicine and Health Sciences, Stellenbosch University and Tygerberg Academic Hospital, Western Cape, South Africa; 4 Department of Biochemistry, Institute of Integrative Biology, Faculty of Health and Life Sciences, University of Liverpool, Crown St, Liverpool, L69 7ZB UK; 5 University College London Hospital NHS Foundation Trust, 250 Euston Road, London, NW1 2PB UK

**Keywords:** Fluorescence imaging, Scanning electron microscopy, Cancer screening, Colorectal cancer, Electron microscopy

## Abstract

Gut dysbiosis contributes to the development of a dysfunctional gut barrier, facilitating the translocation of bacteria and inflammagens, and is implicated in colorectal cancer (CRC) pathogenesis. Such ‘leaky gut’ conditions result in systemic inflammation, of which a hallmark is increased hypercoagulability. Fluorescence antibody confocal microscopy was used to determine circulating levels of lipopolysaccharide (LPS) in control and CRC populations. Here we showed that circulating levels of LPS are significantly elevated in the CRC population. We also showed that markers of inflammation and hypercoagulability are increased in this population. Furthermore, anomalous blood clotting and structural changes in blood components are presented. Importantly, the association between LPS levels, inflammation, and hematological dysfunction was analysed. Statistical regression models were applied to identify markers with strong association with CRC, and to investigate the correlation between markers. A core aim is enhanced biomarker discovery for CRC. We conclude that circulating LPS can promote systemic inflammation and contribute to the development of a pathological coagulation system, with resulting chronic inflammation and an activated coagulation system implicated in tumorigenesis. Blood-based screening tools are an emerging research area of interest for CRC screening. We propose the use of additional (novel) biomarkers to effectively screen for CRC.

## Introduction

Colorectal cancer (CRC) is the fourth most common cancer-related mortality globally, continuing to affect an increasing number of individuals worldwide^1–3^. Novel strategies must therefore be employed to aid in the identification of at risk individuals, considering a combination of possible pathogenetic factors^4^. CRC develops via a complex, multistep interaction between specific genetic susceptibilities and external or environmental stressors^3,5–7^. Hereditary CRCs, such as familial adenomatous polyposis (FAP) and Lynch syndrome, account for less than 5% of CRC cases^8^. Sporadic CRCs are derived from point mutations, affecting the tumour suppressor adenomatous polyposis coli (*APC*) gene (pathway of chromosomal instability)^2^. This mutation stimulates the formation of non-malignant adenomas (polyps), which have the potential for developing into carcinoma. Because the vast majority of CRCs are classified as sporadic (constituting 70% of CRC cases), arising from nonhereditary, spontaneous somatic mutations^2,6,8,9^, the influence of environmental factors in the pathogenesis of CRC is an important consideration when studying this malignancy.

The intestinal microbiota composition is implicated in CRC development, because it impacts immune, structural, and metabolic processes^10,11^. Importantly, changes or disruptions in the composition of the gut microbiota (termed ‘dysbiosis’) can contribute to and form part of colorectal tumorigenesis^2,12,13^. The epithelial cells lining the gut are responsible for early immune responses against pathogens through activation of immune signaling pathways via innate immune receptor molecules (pattern-recognition receptors (PRRs)), thereby maintaining gut homeostasis^10,14,15^. For example, PRR Toll-like receptor 4 (TLR4) has the ability to bind lipopolysaccharide (LPS) from Gram-negative bacteria, which initiates downstream signaling and activates the nuclear transcriptional factor kappa B (NF-κB) signaling pathway, consequently inducing other innate immune responses, pro-inflammatory gene expression, and recruitment of the adaptive immune system^14,15^. Bacterial LPS in the gut strongly stimulates innate immune signaling, thereby compromising gut homeostasis and normal host physiology^16^.

Patients with inflammatory bowel disease (IBD) (Crohn’s disease or ulcerative colitis) have an increased risk (10–15%) for developing colitis-associated CRC^2,5,6,14^. IBD is characterized by a chronic inflammatory state of the intestine, and is associated with a changed gut microbiota composition and the presence of an altered intestinal permeability^14,17^. Moreover, chronic inflammation can in turn impact the composition of the gut microbiota through metabolic changes, ultimately contributing to a dysbiotic state^18^. Therefore, disruptions or alterations to the normal gut flora affect the host immunity (activate the immune system) and chronic inflammation can contribute to the pathogenesis of CRC^2,14,19^. Chronic inflammatory disorders induce gene mutations and stimulate angiogenesis and cell proliferation^20^.

Dysbiosis of the gut contributes to the development of a dysfunctional gut barrier, which facilitates bacterial translocation from the gut^21,22^. Of interest here, is bacterial inflammagens that contribute to systemic inflammation, together with increased hypercoagulability. There is an increased risk of thromboembolic events (hypercoagulability) in IBD, because of defective intestinal barrier functions^17^. Moreover, coagulation dysfunctions (deregulated blood coagulation) frequently occur in cancer patients^23^. Importantly, an activated coagulation system is implicated in the pathogenesis of tumour growth and development, with hypercoagulation being present in most cancer patients^24,25^.

Increased intestinal permeability can lead to persistent systemic inflammation, because intestinal barrier dysfunction allows entry of bacterial products into circulation. The presence of low levels of highly potent circulating inflammagenic molecules such as LPS, results in an increase in inflammatory molecules such as pro-inflammatory cytokines and acute-phase proteins, including serum amyloid A (SAA)^26,27^. Systemic inflammation affects the hematological system by driving coagulopathies (hypercoagulation is an important hallmark of chronic inflammation)^28–30^. Importantly, anomalous clot formation is characterized by the pathologic assembly of fibrin(ogen), containing an amyloid structure^26^. It has been reported that LPS induces fibrin(ogen) to clot into an amyloid form^31,32^. Circulating bacterial inflammagens and inflammatory molecules, such as SAA, have also been shown to generate structural (amyloidogenic) changes in circulating plasma molecules, particularly soluble fibrinogen^33^. This structurally altered conformation in circulating fibrin(ogen) can affect clot formation, thereby contributing to hematological pathology.

This paper investigates the presence of circulating LPS and a set of other circulating biomarkers, indicative of systemic inflammation, in CRC patients. We show that elevated levels of circulating LPS are correlated with dysregulated levels of circulating inflammatory markers. This may, in part, lead to pathological structural changes in blood components, including amyloidogenic and hypercoagulated fibrin(ogen), hyperactivity of platelets, and morphological changes to erythrocytes (RBCs), all contributing to increased hypercoagulability in CRC patients. Therefore, we hypothesize that a bacterial influence, persistent inflammation, hypercoagulation, and colorectal carcinogenesis form an intricate relationship. The use of these markers, either alone or in combination, present an opportunity for improved CRC blood-based screening.

## Methods

### Ethical statement

Ethical clearance for the collection of blood samples from healthy individuals and patients with newly diagnosed colorectal adenocarcinoma was obtained from the Health Research Ethics Committee (HREC) of Stellenbosch University (ethics reference: 6585). All study participants signed an informed consent form prior to sample collection. This study, including sample collection and sample processing, was conducted according to the guidelines set by the Declaration of Helsinki.

### Study population and sample preparation

Blood samples were collected from 41 healthy individuals (16 males and 25 females) with an average age of 51 years, and no history of known inflammatory conditions, chronic disease, chronic medication usage or any anti-inflammatory medication, smoking or oral contraceptive use in the case of females. Whole blood (WB) from healthy individuals was collected in two sodium citrate tubes and a serum separating tube (STT). In addition, blood samples were collected from 26 patients (13 males and 13 females) with newly diagnosed colorectal cancer (CRC), with an average age of 56 years. The stage of all recruited patients was assessed, ranging from 2 to 4, based on histological or imaging tests (CT scans). Of the 26 CRC patients, 10 patients had metastases. None of the patients have had any cancer treatment at the time of sample collection (no neoadjuvant chemotherapy or radiotherapy). Patients may have smoked, could be on some form of chronic medication, with genetic predisposition not forming part of the exclusion criteria. One sodium citrate tube and one SST were used for collection of CRC patient WB. Ultrasensitive C-reactive protein (CRP) and lipid profile assessment of control and CRC blood samples were performed, using SST, at an accredited Pathology Laboratory (PathCare laboratory).

A core aim of this study is biomarker discovery for CRC across a wide range of groups, using WB and platelet poor plasma (PPP). WB in sodium citrate tubes was analysed on the same day of collection, but was first left to stand at room temperature for at least 30 minutes, before thromboelastography (TEG) of WB and scanning electron microscopy (SEM) preparation of WB samples. Citrated WB samples were centrifuged for 15 minutes at 3000 × *g* at room temperature to obtain PPP. The PPP was stored at −80 °C until further sample analyses were performed.

### Vascular injury panel analysis

PPP samples from control and CRC subjects were analysed in duplicate using the V-PLEX Plus Vascular Injury Panel 2 (human) kit from MSD MULTI-SPOT Assay System (K15198G-1). This multiplex kit measures biomarkers that are important in acute inflammation and tissue damage, namely the levels of serum amyloid A (SAA), CRP, soluble vascular cell adhesion molecule-1 (sVCAM-1/CD106) and soluble intercellular adhesion molecule-1 (sICAM-1/CD54). The 96-well plate (pre-coated with capture antibodies) was washed three times with wash buffer, followed by the addition of 25 µL of PPP sample (diluted 1000×), calibrator, or control per well, and incubated for two hours at room temperature. Following another wash step, 25 µL of detection antibody solution (detection antibodies conjugated with electrochemiluminescent labels) was added to each well and incubated for one hour at room temperature. Following the last wash step, read buffer was added to each well. Finally, the plate was loaded into the MSD Discovery Workbench 4 instrument, causing the emission of light by the captured labels. The instrument measures the intensity of the emitted light, which indicates the amount of analyte present in the PPP sample. Biomarker levels are expressed in μg mL^−1^.

### Thromboelastography (TEG) of whole blood (WB) and platelet poor plasma (PPP)

Thromboelastography (TEG) is a method that is used to measure viscoelastic coagulation parameters. Via studying the kinetics of clot formation, the coagulation efficiency (clot formation and clot strength) of WB or PPP samples can be evaluated. TEG analyses were performed on naïve (unexposed/untreated) WB samples and naïve PPP samples, from control subjects and CRC patients. A TEG analysis requires the addition of 340 µL of WB or PPP to 20 µL of 0.2 mol L^−1^ activator calcium chloride (CaCl_2_) in a disposable TEG cup. The addition of CaCl_2_ reverses the effect of the sodium citrate anticoagulant in the collection tube, thereby initiating clotting/coagulation. The samples were placed in a computer-controlled Thromboelastograph 5000 Hemostasis Analyzer System for analysis at 37 °C, configured and used according to the manufacturer’s protocol.

### Scanning electron microscopy (SEM) analysis of whole blood (WB) smears and platelet poor plasma (PPP) clots

Control and CRC WB were prepared for scanning electron microscopy (SEM) analysis by placing 15 μL directly onto 10 mm round glass cover slips, followed by slightly smearing the blood drop across the surface of the cover slip. SEM preparation of CRC WB samples was performed in a dead-air hood (with ultraviolet light exposure prior to sample preparation). WB smears were allowed to dry for ±3 minutes at room temperature, to allow the cells to adhere to the glass slips. In addition to study the ultrastructure and morphology of RBCs and platelets, SEM was also used for the ultrastructural analysis of control and CRC PPP clots (to assess and compare fibrin network structure). For fibrin network analysis, 5 μL of thrombin, provided by the South African National Blood Service, was added to 10 μL PPP (at 7 U mL^−1^) on glass cover slips. Thrombin activates fibrin polymerisation (converts fibrinogen to fibrin) and creates extensive fibrin fibre networks. All cover slips were then placed in 24-well plates. WB smears were subsequently washed in Gibco phosphate-buffered saline (PBS) (pH = 7.4) (ThermoFisher Scientific, 11594516) for 15 minutes. PPP clots were immediately submerged in PBS for ±20 minutes. All samples were fixed in 4% paraformaldehyde for a minimum of 30 minutes (to crosslink proteins), followed by three PBS wash steps. Samples then underwent secondary fixation with 2–3 drops of 1% osmium tetroxide (OsO_4_) (Sigma-Aldrich, 75632) in double distilled H_2_O for an additional 15–30 minutes. Fixation with OsO_4_ stabilises membranes and ultrastructural features, enhances contrast, and provides conductivity under the scanning electron beam. Following post-fixation, samples were washed three times in PBS. The samples were then gradually dehydrated with increasing concentrations of ethanol for 3 minutes each (30%, 50%, 70%, 90%, and then three times with 100% ethanol). Sample dehydration was completed with 99.9% hexamethyldisilazane (HMDS) ReagentPlus (Sigma-Aldrich, 379212) treatment for 30 minutes. The HMDS was removed and replaced with one final drop of HMDS, directly placed onto the samples, after which the samples were left to air-dry in a fume hood overnight (±16 hours). Dried samples were mounted on glass microscope slides with double-sided carbon tape, and then sputter coated with a thin (±5 nm thick) layer of carbon prior to SEM imaging, using a Quorum Q150T coater by performing carbon rod evaporation. This enhances conductivity, by preventing build-up of high-voltage charges. SEM ultrastructural analysis of WB smears and PPP clots was performed on the Zeiss MERLIN field emission scanning electron microscope, located in the Central Analytical Facility (CAF) Electron Microbeam Unit, Stellenbosch University. All micrographs were captured using the InLens detector at 1 kV electron beam.

In addition to ultrastructural (qualitative) analysis of PPP clots, changes in fibrin fibre diameter between control and CRC PPP clots were quantified. Fibrin networks of ten control samples and ten CRC samples (age- and gender-matched) were analysed, captured at 10000x machine magnification. A representative micrograph of each sample was selected and a 1 × 1 μm grid (scaled according to the machine magnification) was overlaid over these micrographs using ImageJ (FIJI). We analysed 8 × 8 blocks (an area of 8 × 8 μm) and one fibre was randomly selected for measurement (in nanometers) per block, which resulted in 64 measurements per micrograph. Thick fibres visible as laterally fused fibres were treated as a single fibrin fibre. In cases where a block did not contain any visible individual fibres, but only displayed a hypercoagulable dense matted mass, the diagonal length of the block (maximum fibre diameter of 248 nm) was allocated. This systematic approach ensured that the fibres were measured in an unbiased manner, and that a variety of different fibres in the selected area was included for measurement. Micrographs were enlarged to an extent in order to easily measure the diameters of thin fibres. Frequency histograms were constructed in GraphPad Prism 7.04, to graphically display fibre diameter distribution. Moreover, the average fibrin fibre diameter, maximum fibrin fibre diameter, and minimum fibrin fibre diameter of the ten control samples and ten CRC samples were noted.

### Immunostaining of platelet poor plasma (PPP) smears

PPP (5 µL) from healthy subjects and CRC patients was smeared on a microscope slide, after which the slide was air dried for ±45 minutes. The sample was then fixed with 10% neutral buffered formalin (NBF) for 3–5 minutes. Following three PBS washes, the sample was blocked with 5% goat serum (prepared in PBS) for 30 minutes, after which it was stained with primary antibody (made up in 5% goat serum in 1:200), Anti-*E. coli* LPS antibody [2D7/1] (mouse monoclonal IgG, ab35654, Abcam), for one hour at room temperature. This primary antibody was used as a proxy to measure the levels of the Gram-negative membrane component LPS. Lipid A is a highly conserved diglucosamine-based phospholipid^34^, and the lipid A moiety synthesized by most Gram-negative bacteria is similar to (or resembles) the lipid A molecule of *E. coli*^35,36^. Following three PBS washes, the sample was stained with secondary antibody (made up in PBS in 1:200), Goat Anti-Mouse IgG H&L (Alexa Fluor 488) (ab150113, Abcam), for one hour at room temperature in the dark. Finally, following the last wash step, a cover slip was mounted with a drop of Dako fluorescence mounting medium for confocal analysis. Prepared PPP smears were stored at −20 °C (protected from light), and viewed using a Zeiss LSM 780 with ELYRA PS1 confocal microscope with a Plan-Apochromat 63×/1.4 Oil DIC M27 objective. A 488 nm Argon excitation laser was used, with emission measured at 493–630 nm. Gain settings were kept constant for data acquisition, to permit accurate statistical analyses. Of each prepared PPP smear, 4–8 micrographs were captured, as well as from secondary antibody controls. Non-stained controls were also included. Of 29 control PPP smears, 145 images were acquired, and 154 images from 25 CRC PPP smears. The mean fluorescence intensity (MFI) from all acquired images was computed (using ImageJ (FIJI)^37^), followed by dividing the MFI of each image by the average MFI of the corresponding secondary antibody control images, resulting in normalised fluorescence intensity values per control or CRC sample. In this manner, by accounting for non-specific secondary antibody binding, anti-*E. coli* LPS antibody binding could be accurately quantified.

### Confocal analysis of platelet poor plasma (PPP) clots

In order to investigate the fluorescence (anomalous) amyloid signal present in control and CRC PPP clots, PPP samples were incubated with the amyloid-selective Amytracker 630 marker (Ebba Biotech AB). Control and CRC PPP were prepared for confocal analysis by adding 2 µL of the fluorescent amyloid marker (of the working solution, made up by diluting the stock concentration in PBS at a 1:20 ratio) into 96 μL PPP. Following the addition of Amytracker 630, it was incubated in the plasma for 30 minutes (protected from light) at room temperature. In addition, unstained PPP of healthy individuals and CRC patients was also included for analysis, to investigate the presence of autofluorescence signal in unstained control and CRC PPP clots. After incubation time (for stained samples), PPP clots were created by transferring a small volume (10 µL) of the stained (or unstained) PPP sample to a microscope slide, after which thrombin at 7 U mL^−1^ was added in a 1:2 ratio (5 µL thrombin: 10 µL PPP), to create extensive fibrin fibre networks. A cover slip was placed over the prepared clot, and control and CRC clots were viewed using a Zeiss LSM 780 with ELYRA PS1 confocal microscope with a Plan-Apochromat 63×/1.4 Oil DIC M27 objective. A 488 nm Argon excitation laser was used, with emission measured at 579–695 nm. For data acquisition and comparison between control and CRC clot images, gain settings were kept constant to ensure a reliable statistical outcome. All micrographs of the prepared clots were captured in an unbiased manner as 3 × 3 tile images, with 3–5 tiles captured per sample. The MFI from all acquired PPP clot images was computed (using ImageJ (FIJI)) to compare the response to the Amytracker 630 stain in control and CRC populations, as well as for comparing unstained clots.

### Statistical analysis

To determine the strength of associations between biomarkers and CRC, logistic and ordinal regression were performed. Three models were compared: model 1 uses unadjusted logistic regression, model 2 adjusts for age and gender, and model 3 considers an ordinal relationship between the biomarkers and CRC stage (ranging from 2 to 4). The adjustment addresses the small differences between the control and CRC age and gender distribution – removing these possible confounding factors. Ordinal regression was performed to investigate the strength of the association between biomarker levels and the stage of CRC (i.e. disease progression). Furthermore, linear regression was also performed to determine the correlation between selected parameters. Statistical significance was accepted at p < 0.002 (with level adjusted based on Bonferroni correction). This statistical analysis was performed using R version 3.6.1 using the stats and ordinal packages. GraphPad Prism 7.04 was also used to prepare image plots and perform the corresponding unpaired t-tests or Mann-Whitney non-parametric tests.

## Results

### Systemic inflammation, altered lipid metabolism, and vascular dysfunction

Table 1 shows the sample demographics, Pathology Laboratory results, as well as the V-PLEX Plus Vascular Injury Panel 2 (human) kit results of both populations. The significantly higher ultrasensitive CRP levels in CRC patients, compared to age-matched controls, illustrate the presence of active infection/inflammation in these patients. The logistic and ordinal regression models indicated that this parameter is statistically significant, being predictive of an increased chance of CRC and also correlating to the stage of CRC. PPP samples from healthy individuals and CRC patients were analysed for specific inflammatory biomarkers, using the vascular injury panel kit. It is known that the non-specific hepatic inflammatory markers CRP and SAA are persistently elevated during chronic inflammation, as a result of infectious stimuli or trauma^38^. CRP plasma levels can increase from ±1 μg mL^−1^ to over 500 μg mL^−1^ within 24–72 hours of severe tissue damage, including those caused by trauma and progressive cancer^39^. CRP levels are significantly increased in CRC patients, indicative of local inflammation, also acting as marker during a systemic inflammatory response^40,41^. Both the logistic and ordinal regression models identified this parameter as statistically significant, suggesting that CRP levels are associated with CRC and correlates to CRC stage/progression. The serum/plasma SAA concentration in healthy individuals range from 1–5 μg mL^−1^. However, under pathological conditions, including inflammatory or infectious diseases, as well as neoplasia, serum SAA concentrations >10 μg mL^−1^, and up to 1 mg mL^−1^ can be found^42^. The SAA levels are also significantly elevated in CRC patients, with SAA identified as a significant parameter across all models. Moreover, CRC patients show significantly upregulated levels of the pro-thrombotic mediator sICAM-1, as observed in a variety of inflammatory conditions^43^. Healthy sICAM-1 serum concentrations have been reported to range between 0.1 μg mL^−1^ and 0.408 μg mL^−1^ ^44^. This parameter is identified as significant across all models, but there is not a significant difference in sVCAM-1 levels between control and CRC populations.

**Table 1:** Demographics, Pathology Laboratory results, and vascular injury panel results of healthy individuals and colorectal cancer (CRC) patients.

Demographics						
	**Healthy individuals**			**CRC patients**		
**Gender**	Male (n = 16), Female (n = 25)			Male (n = 13), Female (n = 13)		
**Age (years)**	50 [44–56.5]			56.5 [46.25–67.5]		
**Pathology Laboratory results**						
	**Reference (normal)**	**Healthy individuals (n** = **40)**	**CRC patients (n** = **26)**	**Unadjusted OR (99.8% CI)**	**Adjusted OR (99.8% CI)**	**Ordinal OR (99.8% CI)**
**Total cholesterol (mmol L**^**−1**^**)**	**<5.0**	5.3 [4.6–5.8]	4 [2.98–5.08]	**0.42076 (0.16054–0.84944)**	**0.41326 (0.14649–0.86124)**	**0.45806 (0.21885–0.85567)**
**HDL cholesterol (mmol L**^**−1**^**)**	**>1.2**	1.4 [1.2–1.6]	0.9 [0.73–1.08]	**0.0036712 (1.6763** × **10**^−**5**^–**0.11499)**	**0.00070242 (2.5039** × **10**^−**7**^–**0.063268)**	**0.013985 (0.00045301–0.18081)**
**LDL cholesterol (mmol L**^**−1**^**)**	**<3.0**	3.2 [2.5–3.8]	2.55 [1.65–3.35]	0.50688 (0.1926–1.0978)	0.51151 (0.18674–1.1181)	0.50247 (0.2148–1.0681)
**Triglyceride (mmol L**^**−1**^**)**	**<1.70**	1.37 [0.95–1.94]	1.45 [0.95–1.69]	0.61177 (0.14169–1.7216)	0.52288 (0.1038–1.5875)	0.6475 (0.16181–1.7703)
**Non-HDL cholesterol (mmol L**^**−1**^**)**	**<3.8**	3.8 [3.1–4.6]	3.25 [2.33–4]	0.55199 (0.22654–1.1447)	0.55252 (0.22059–1.1556)	0.54693 (0.245–1.1199)
**Total cholesterol/HDL cholesterol ratio**	**<4.0** >4 – moderate risk >5 – high CVD risk	3.7 [3.2–4.3]	4.6 [4.13–5.48]	1.9225 (0.99881–5.0375)	**2.0944 (1.0311–5.7913)**	**1.853 (1.0502–3.7879)**
**Ultrasensitive CRP (mg L**^**−1**^**)**	**<1** <1 – low risk 1–3 – moderate risk >3 – high risk >5.0 – consider active infection/inflammation	1.6 [0.74–3.18]	56.77 [8.41–119.77]	**1.9276 (1.1709–5.0789)**	**1.9885 (1.1844–5.2185)**	**1.0173 (1.0045–1.0333)**
**Vascular injury panel results**						
**Biomarker**	**Reference (normal)**	**Healthy individuals (n** = **40)**	**CRC patients (n** = **26)**	**Unadjusted OR (99.8% CI)**	**Adjusted OR (99.8% CI)**	**Ordinal OR (99.8% CI)**
**SAA (μg mL**^**−1**^**)**	**1–5**	1.55 [0.77–2.51]	25.5 [5.85–256.23]	**1.041 (1.008–1.1663)**	**1.04 (1.0081–1.1594)**	**1.0091 (1.0014–1.0178)**
**CRP (μg mL**^**−1**^**)**	**<1**	1.34 [0.55–3.06]	56.19 [8.28–125]	**1.1537 (1.0311–1.5018)**	**1.1459 (1.0297–1.4963)**	**1.0232 (1.0082–1.0414)**
**sVCAM-1 (μg mL**^**−1**^**)**		0.39 [0.31–0.45]	0.43 [0.34–0.54]	141.85 (0.43296–293214)	104.95 (0.21565–319110)	135.44 (0.7473–50350)
**sICAM-1 (μg mL**^**−1**^**)**	**0.1–0.408**	0.29 [0.24–0.34]	0.56 [0.38–0.76]	**400925647 (1274.2–1.2605** × **10**^**19**^**)**	**1230573039 (1465.2–1.7109** × **10**^**20**^)	**151.71 (6.1794–14694)**

Data expressed as median and [25−75% quartile range]. Significant differences are shown in bold for all models. All CRC patients with hypertension used medication for blood pressure regulation. Prescribed medication was recorded, and included Enalapril, Ridaq, Atenolol, Amlodipine, LASIX (furosemide), Losartan, and Perindopril.

Inflammation and infections result in abnormalities in lipid metabolism, classically associated with elevated levels of serum total cholesterol, low-density lipoprotein (LDL) cholesterol, triglycerides, and decreased levels of serum high-density lipoprotein (HDL) cholesterol^45^. CRC patients have significantly decreased serum total cholesterol and HDL cholesterol levels, compared to healthy individuals. These parameters are identified as significant across all models. However, LDL cholesterol levels, non-HDL cholesterol levels, and triglyceride levels are not predictive of an increased chance of CRC (or associated with CRC stage). When adjusting for age and gender, some markers showed a *stronger* association with CRC. For example, after adjustment for age and gender, there is stronger association between a decrease in HDL cholesterol levels and the odds of CRC. The lipid profile of CRC patients illustrates an altered lipid metabolism, but not hyperlipidaemia. Moreover, the total cholesterol/HDL cholesterol ratio, an indicator of cardiovascular disease (CVD) risk, is identified as a significant parameter by logistic regression (when adjusted for age and gender) and ordinal regression models. In summary, CRC patients demonstrate a dysregulated lipid metabolism, together with a pro-inflammatory and pro-thrombotic state.

### Hypercoagulability and pathological structural changes in blood components

**Figure 1:**
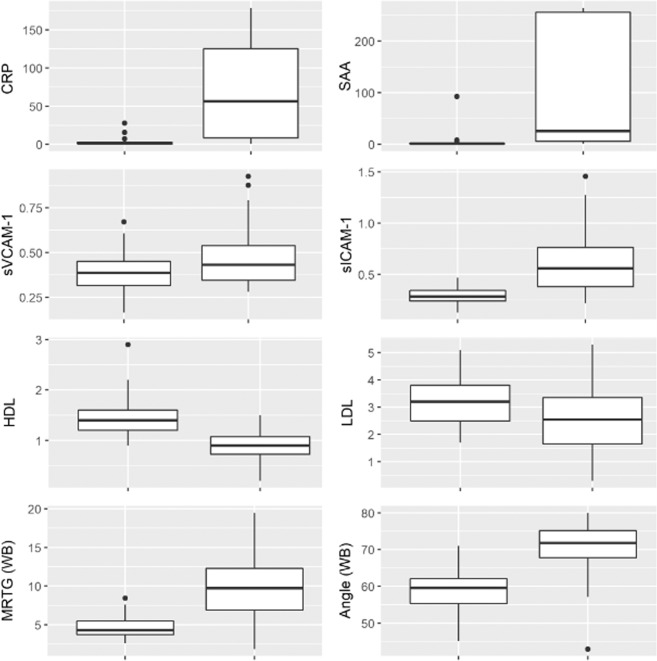
Box and whisker plots of the four vascular injury panel biomarkers (μg mL^−1^), HDL cholesterol and LDL cholesterol levels (mmol L^−1^), and the TEG WB clot parameters MRTG (Dyn cm^−2^ s^−1^) and Angle (degrees) for healthy individuals (left box) and colorectal cancer (CRC) patients (right box).

The hematological system is sensitive and highly responsive to dysregulated (upregulated) circulating inflammatory molecules, which affect the morphology, ultrastructure, and function of platelets, RBCs, and fibrin(ogen), all influencing (hyper)coagulability. Clotting parameters, as measured by TEG, indicate to what degree the coagulation system is activated (or pathological) over time. The results of the TEG analysis of CRC WB are shown in Table 2. No significant difference was observed in the clot initiation time/reaction time (R value) between controls and CRC patients. However, compared to healthy individuals, CRC patients showed a significantly decreased time (or increased rate) to achieve a certain level of clot strength (amplitude of 20 mm), indicated by kinetics (K value). CRC patients also had significantly higher TEG values for the clot angle (reflecting the rate of fibrin fibre cross-linking or clot formation/thrombin burst), as well as for the maximum amplitude (MA) (maximum strength/rigidity/stiffness of the clot). Moreover, the maximum velocity of clot growth (MRTG) and total clot strength (TTG) were significantly increased in CRC patients. However, there was not a significant difference in the time from clot initiation to maximum velocity of clot growth (TMRTG) between controls and CRC patients. Specifically, changes in MRTG and TTG reflect changed or abnormal polymerization of the soluble plasma protein fibrinogen to insoluble fibrin fibres^46^. The other coagulation or clotting parameters are indicative of the interactions between all blood components, including cells and plasma proteins, influencing coagulation^46^. In summary, of the seven viscoelastic parameters used to assess coagulation efficiency, five coagulation parameters differed significantly between healthy controls and CRC patients. Both the logistic and ordinal regression models identified these parameters as significant, having a strong association with (predictive of an increased chance of) CRC and also correlates to CRC stage. The two TEG WB clot parameters that did not illustrate a clinical significance are related to clot initiation time (R value) and time taken to achieve maximum velocity of clot growth (TMRTG). However, once initiated, a clot of increased strength forms faster. Together, these differences suggest an increased clotting potential of WB from CRC patients, compared to WB of healthy individuals. Fig. 1 shows box and whisker plots of the four inflammatory biomarkers of the vascular injury panel, HDL cholesterol and LDL cholesterol levels, as well as the two most significant TEG WB clot parameters (MRTG and Angle).

**Table 2:** Results of the seven viscoelastic thromboelastography (TEG) clot parameters assessing (hyper)coagulability of whole blood (WB) and platelet poor plasma (PPP) samples from healthy individuals and colorectal cancer (CRC) patients.

**TEG WB clot parameter**	**Normal range**	**Healthy individuals (n** = **28)**	**CRC patients (n** = **26)**	**Unadjusted OR (99.8% CI)**	**Adjusted OR (99.8% CI)**	**Ordinal OR (99.8% CI)**
**Reaction time (R value)**	9–27 min	8.05 [6.92–10.05]	7.8 [6.43–8.78]	0.87941 (0.59391–1.2433)	0.87392 (0.57785–1.2464)	0.91336 (0.65321–1.2483)
**Kinetics (K value)**	2–9 min	2.9 [2.7–3.7]	1.65 [1.3–2.08]	**0.26064 (0.05417–0.76744)**	**0.24436 (0.047616–0.75816)**	**0.29566 (0.083756–0.76564)**
**Angle (A/Alpha)**	22–58°	59.6 [55.3–62.1]	71.8 [67.8–75.2]	**1.2317 (1.068–1.5303)**	**1.245 (1.0732–1.561)**	**1.1859 (1.0573–1.3768)**
**Maximum amplitude (MA)**	44–64 mm	58.4 [54.9–62]	70.1 [64.2–74]	**1.1224 (1.0158–1.2952)**	**1.1268 (1.0178–1.3069)**	**1.1128 (1.0189–1.2447)**
**Maximum rate of thrombus generation (MRTG)**	0–10 Dyn cm^−2^ s^−1^	4.29 [3.7–5.5]	9.73 [6.9–12.3]	**2.0366 (1.2755–4.728)**	**2.153 (1.3059–5.1693)**	**1.3702 (1.1001–1.8041)**
**Time to maximum rate of thrombus generation (TMRTG)**	5–23 min	11.2 [10.1–15]	10.9 [8.7–12.7]	0.87626 (0.65129–1.1299)	0.87747 (0.64541–1.1412)	0.89863 (0.69455–1.1313)
**Total thrombus generation (TTG)**	25–1014 Dyn cm^−2^	703 [611–819]	1181 [904–1437]	**1.0041 (1.0011–1.0089)**	**1.0042 (1.0011–1.0093)**	**1.0028 (1.0007–1.0053)**
**TEG PPP clot parameter**		**Healthy individuals (n** = **39)**	**CRC patients (n** = **26)**	**Unadjusted OR (99.8% CI)**	**Adjusted OR (99.8% CI)**	**Ordinal OR (99.8% CI)**
**R value**		8.9 [7.85–11.2]	8.4 [6.48–10.35]	0.91619 (0.69394–1.1522)	0.87447 (0.64143–1.1144)	0.92781 (0.70967–1.1693)
**K value**		3.3 [2.5–4.9]	1.45 [1.13–2.3]	0.71491 (0.39003–1.0604**)**	0.72711 (0.40712–1.0595)	0.71052 (0.39006–1.0728)
**A**		56.2 [52.1–64.3]	74.2 [65.5–77.4]	**1.2109 (1.0774–1.4523)**	**1.2072 (1.0717–1.4533)**	**1.1738 (1.0665–1.3287)**
**MA**		25.5 [22.1–30.2]	38.4 [30–45]	**1.2333 (1.0807–1.5199)**	**1.2265 (1.0723–1.5132)**	**1.1132 (1.0309–1.2277)**
**MRTG**		3.51 [2.72–4.33]	8.65 [5.38–11.9]	**1.8801 (1.2536–3.6609)**	**1.7994 (1.2214–3.4995)**	**1.1604 (1.011–1.4374)**
**TMRTG**		10.8 [8.92–12.6]	9.84 [7.25–12]	0.9413 (0.73766–1.1502)	0.90251 (0.68618–1.1159)	0.95088 (0.74862–1.1649)
**TTG**		171 [142–217]	312 [214–411]	**1.0194 (1.0069–1.0402)**	**1.0189 (1.0062–1.0398)**	**1.0059 (1.0009–1.0131)**

Data expressed as median and [25−75% quartile range]. Significant differences are shown in bold for all models.

**Figure 2:**
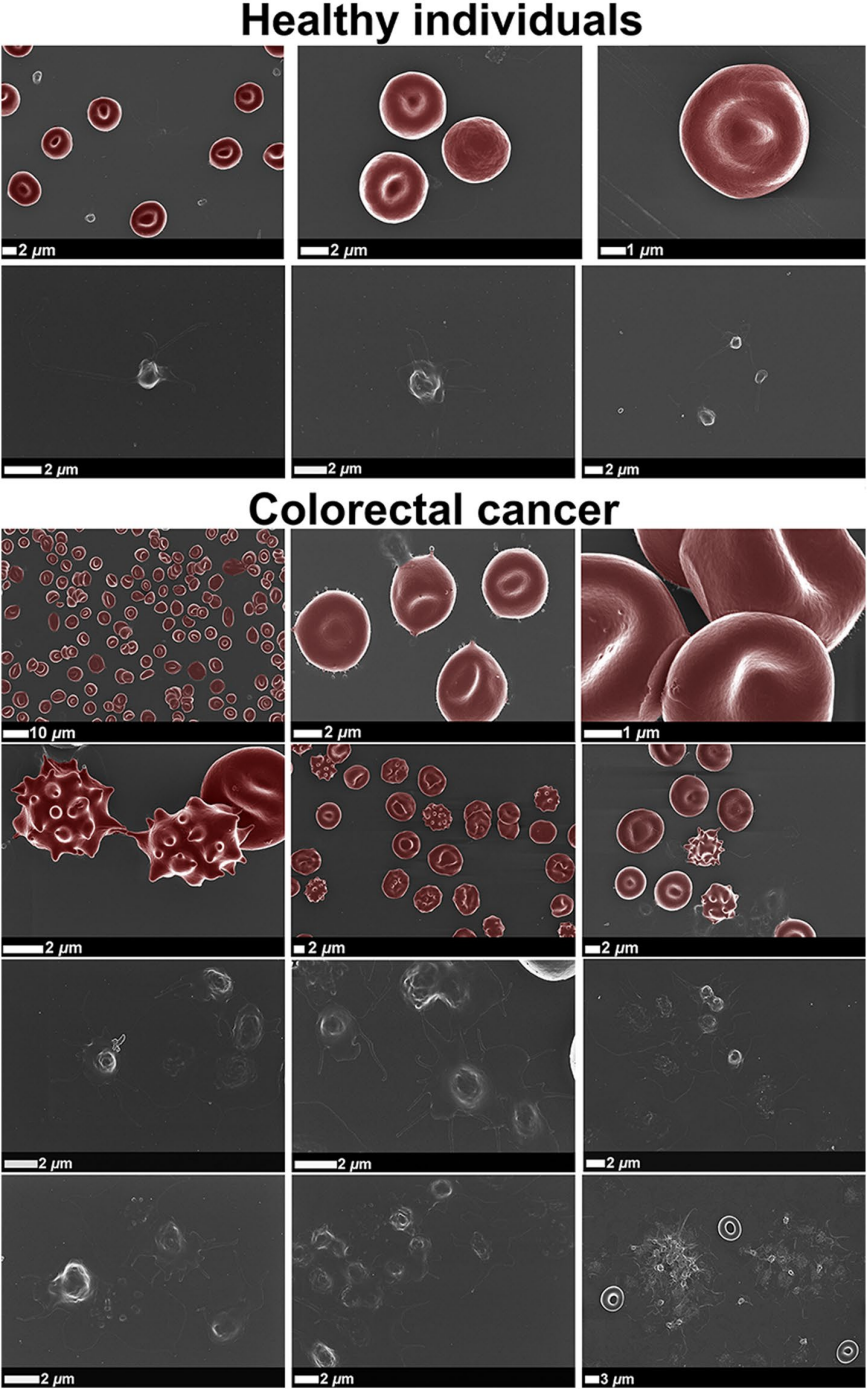
Representative scanning electron micrographs of whole blood (WB) smears, focusing on erythrocytes (RBCs) and platelets of healthy individuals and colorectal cancer (CRC) patients.

Representative SEM micrographs of WB smears from healthy individuals and CRC patients are shown in Fig. 2. Healthy RBCs illustrate a typical discoid shape, whereas RBCs from CRC patients display atypical/abnormal non-discoid shapes, with increased number of eryptotic RBCs. Furthermore, healthy RBC membranes present smooth membrane surfaces, while CRC patients display pathological changes to RBC membrane structure. RBC dysfunction may potentially lead to anaemic conditions in CRC patients. RBC aggregates appear frequently in WB smears from CRC patients. Platelets from healthy individuals have a small and round appearance, only slightly activated (displaying pseudopodia formation), due to contact activation during sample preparation. However, an increased level of platelet activation, membrane spreading, as well as platelet-derived microparticle formation are present in WB smears from CRC patients. Platelet aggregates or platelet clumping are also abundantly present in CRC WB. Importantly, the aim here was not to indicate differences in samples from patients with different stages of CRC, but to compare SEM micrographs of WB smears from healthy individuals to that from CRC patients as a group (ranging from stage 2 to 4). All CRC patients, independent of the stage, demonstrate comparable pathological changes to the morphology and ultrastructure of blood cells, because all CRC patients are burdened with systemic inflammation. An activated coagulation system is a hallmark of persistent inflammation. SEM micrographs of WB smears from all CRC patients therefore show similar pathological features, indicative of increased hypercoagulability.

**Figure 3:**
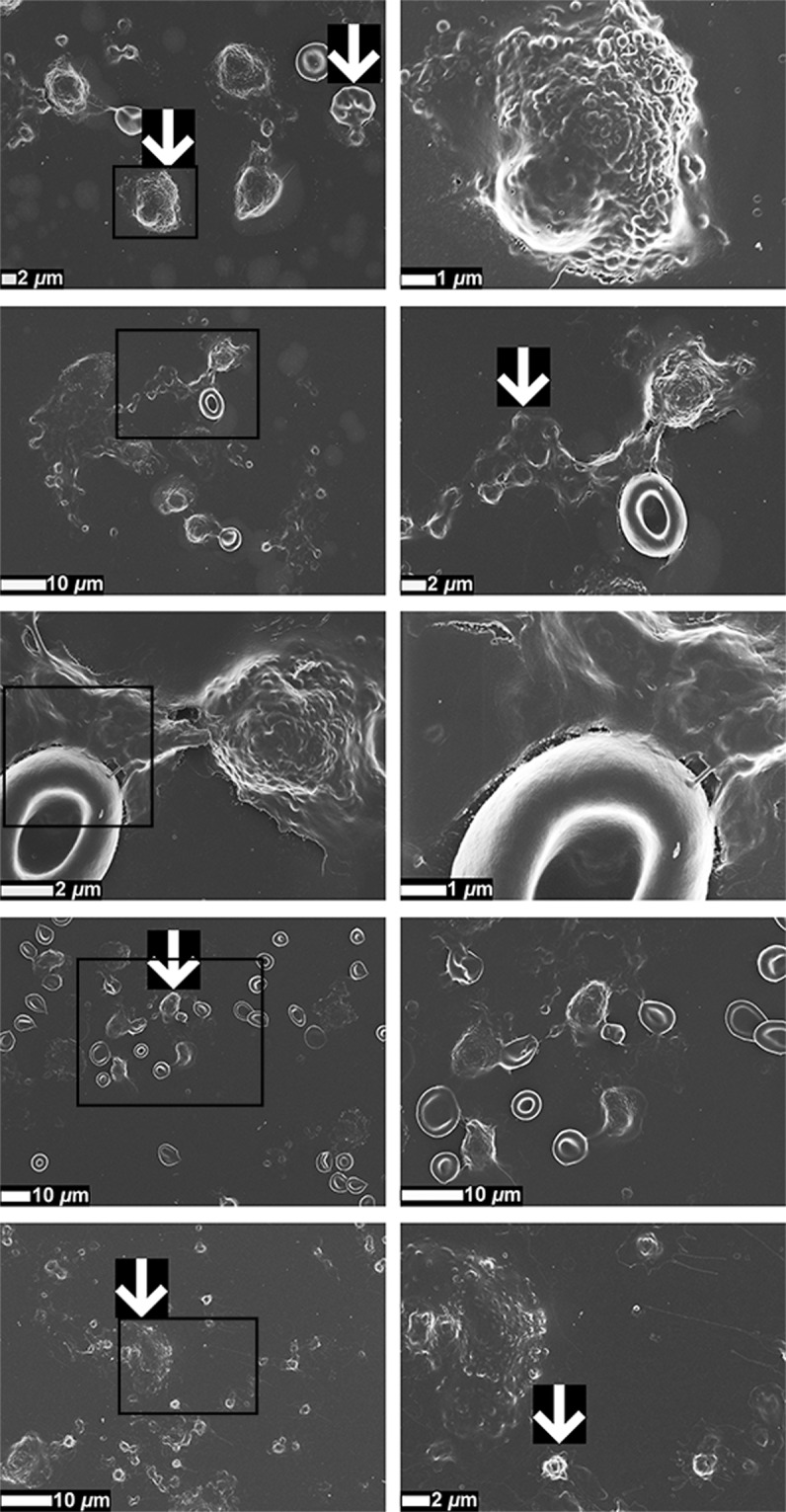
Representative scanning electron micrographs of whole blood (WB) smears of stage 4 colorectal cancer (CRC) patients, focusing on different cellular interactions. White arrows indicate hyperactivated platelets, eryptotic erythrocytes (RBCs), leukocytes, and possible circulating tumour cells (CTCs). Micrographs in the second column are higher magnification (zoomed-in) micrographs of the areas indicated with black boxes in the first column.

Representative SEM micrographs of WB smears from only stage 4 CRC patients are shown in Fig. 3, focusing on the interactions between different blood components (platelets, RBCs, leukocytes, and possibly cancer cells). It is known that circulating tumour cells (CTCs) can have different sizes (have a large heterogeneity), but most have large sizes^47^. However, CTCs may also have similar sizes than leukocytes. It is therefore difficult to identify cancer cells in the presence of other blood components with SEM analysis. However, Fig. 3 illustrates abundant agglutination between various cellular entities in stage 4 CRC patients, which may influence hypercoagulability.

**Figure 4:**
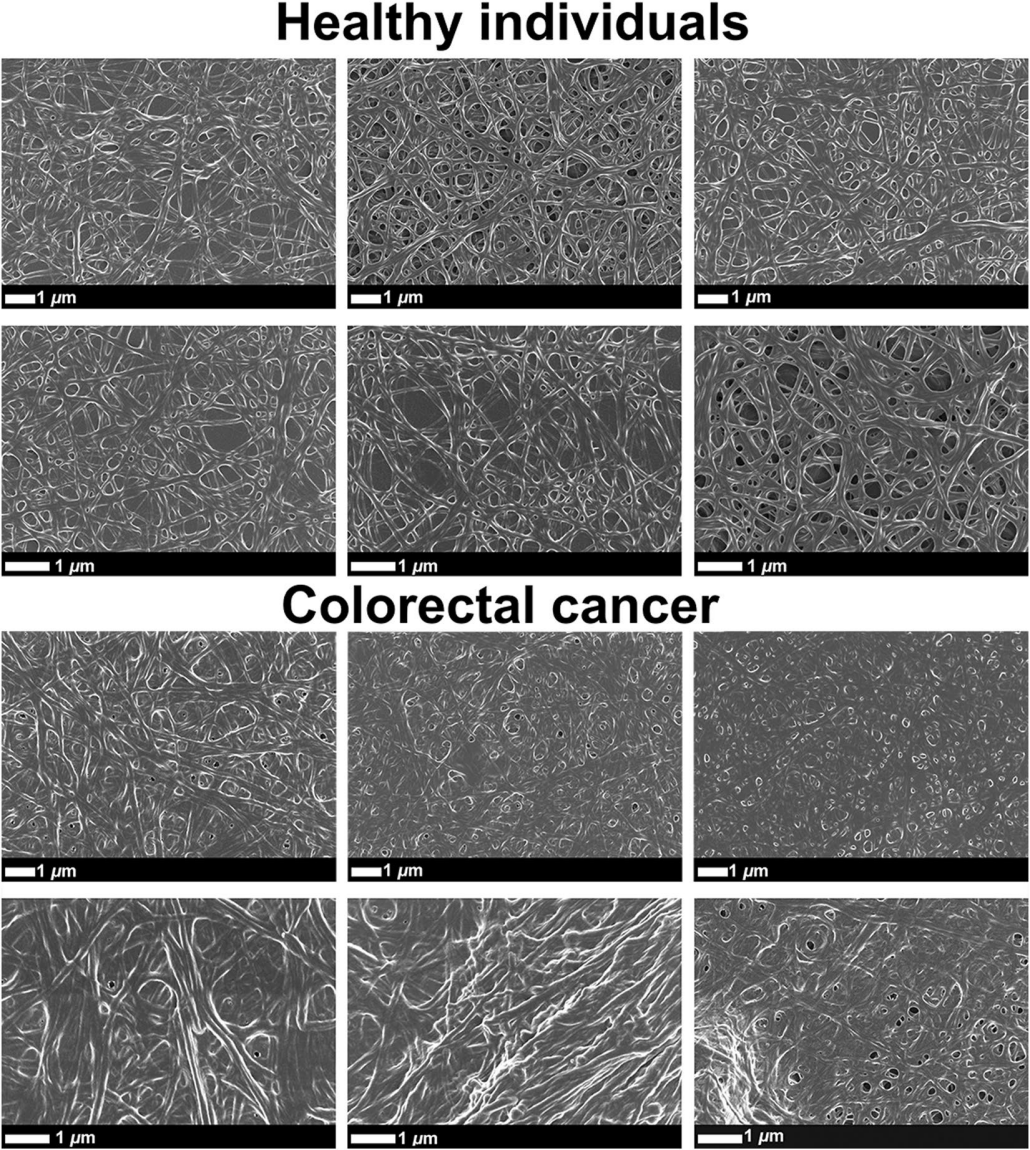
Representative scanning electron micrographs of fibrin clots from healthy individuals and colorectal cancer (CRC) patients, where thrombin was added to platelet poor plasma (PPP) to create extensive fibrin networks. Micrographs were enhanced for publication clarity, by adjusting the brightness and contrast of the images in Adobe Photoshop CS6.

Similar significant differences were observed when comparing the kinetic changes of CRC PPP to that of healthy controls, as seen with TEG analysis of CRC WB (refer to Table 2). Four coagulation parameters out of the seven viscoelastic TEG clot parameters were identified as significant across all models. The changes observed in TEG PPP clot parameters, mainly affected by fibrin(ogen), suggest that the rate of clot formation and clot strength are increased in CRC patients. Importantly, clot ultrastructure (an important way to measure hematological healthiness) of healthy PPP clots and CRC PPP clots can be assessed via SEM analysis, which may convey important visual information regarding changes in fibrin packaging, involved in the clotting process^48^. Fig. 4 shows representative SEM micrographs, indicating the structural differences between fibrin fibre networks from healthy clots and CRC clots. Clot structure of healthy PPP (with added thrombin) appears as looser fibrin networks, with more open spaces visible between branching fibres. It is typical of healthy fibrin clots to form a net where individual fibrin fibres are distinguishable. However, PPP from CRC patients, with added thrombin, appears as a meshed network (or dense matted deposits). Thicker fibres are fused together, and cannot always be distinguished. When thick/dense plates are observed, it is an indication of hypercoagulability.

**Figure 5:**
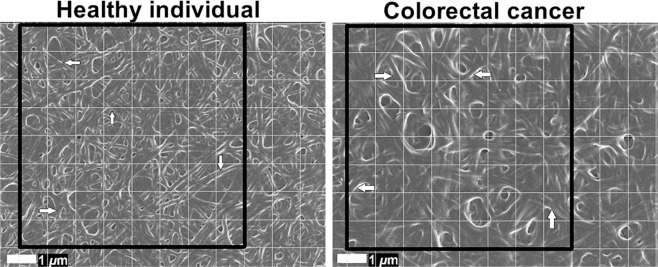
Scanning electron micrographs (overlaid with a 1 × 1 μm grid) of fibrin clots from a healthy individual and colorectal cancer (CRC) patient, where thrombin was added to platelet poor plasma (PPP) to create extensive fibrin networks. Arrows indicate examples of fibres selected for measurement in blocks. Micrographs were enhanced for publication clarity, by adjusting the brightness and contrast of the images in Adobe Photoshop CS6.

**Figure 6:**
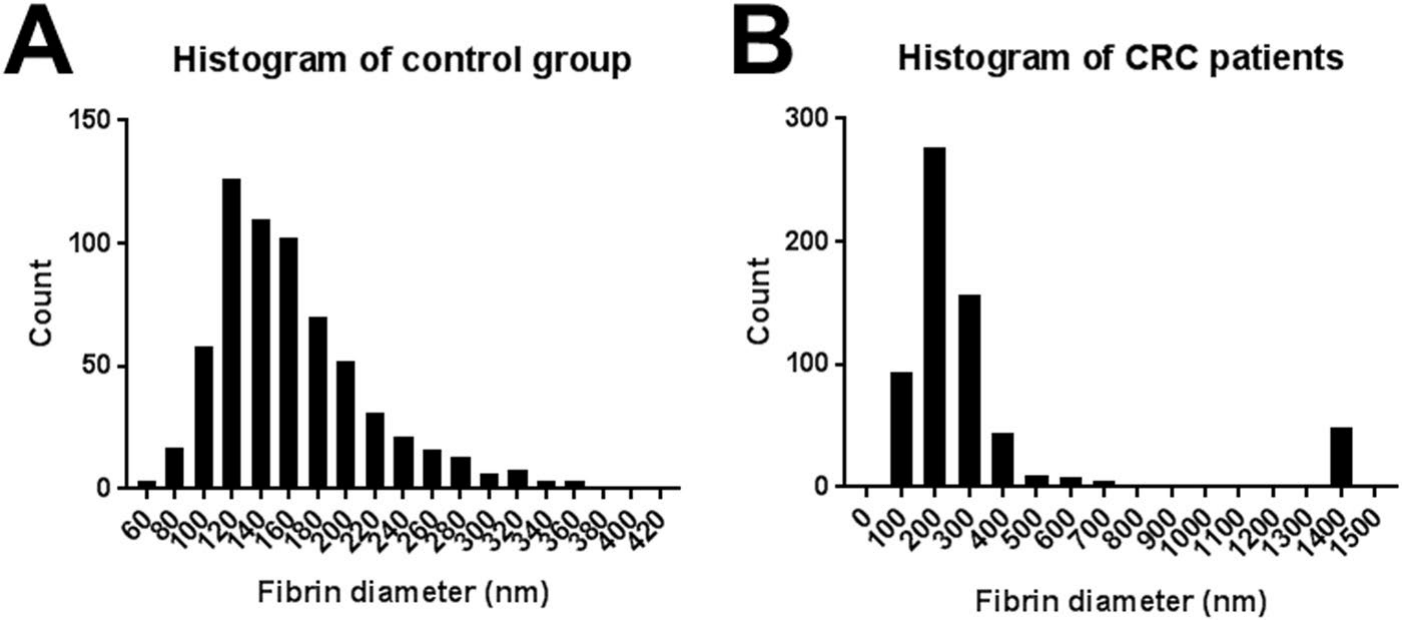
Histograms illustrating the frequency distributions of the fibrin fibre diameters of fibrin clots from (**A**) healthy individuals (n = 10) and (**B**) colorectal cancer (CRC) patients (n = 10).

In Fig. 5, a 1 × 1 μm grid is overlaid on micrographs of representative fibrin clots of the control group and CRC patient group (with the 8 × 8 μm areas indicated by the black squares), where white arrows indicate examples of fibres that were selected for diameter measurement. Histograms of the frequency distributions of fibre diameters of the control group and CRC patients are shown on Fig. 6. The single largest and smallest measurement of each healthy individual were compared to the single largest and smallest measurement of each CRC patient. Similarly, the average fibre diameter of each healthy individual was compared to the average fibre diameter of each CRC patient (refer to Table 3). It is clear from Fig. 6 that healthy fibrin fibres have smaller diameters, distributed between a range of 60–360 nm. On the other hand, fibrin fibres in CRC PPP clots have a more compact distribution (over larger fibrin diameters), which results in higher counts of larger diameters. The average, maximum, and minimum diameters of fibrin fibres in clots created from CRC PPP are significantly higher than that from healthy PPP.

**Table 3:** Analysis of fibrin fibre diameters of healthy individuals (n = 10) and colorectal cancer (CRC) patients (n = 10), with the corresponding significance (***p < 0.001; ****p < 0.0001).

**Fibrin fibre diameter (nm)**	**Control group**	**CRC group**	**Significance**
**Average**	158.9 [145.3–176.3]	339.8 [311.2–351.8]	****
**Maximum**	332.4 [295.5–353.1]	1418 [1256–1418]	****
**Minimum**	81 [68.77–93.36]	110.7 [98.73–150.8]	***

Data expressed as median and [25–75% quartile range].

### Bacterial lipopolysaccharide (LPS) in the circulation of CRC patients

**Figure 7:**
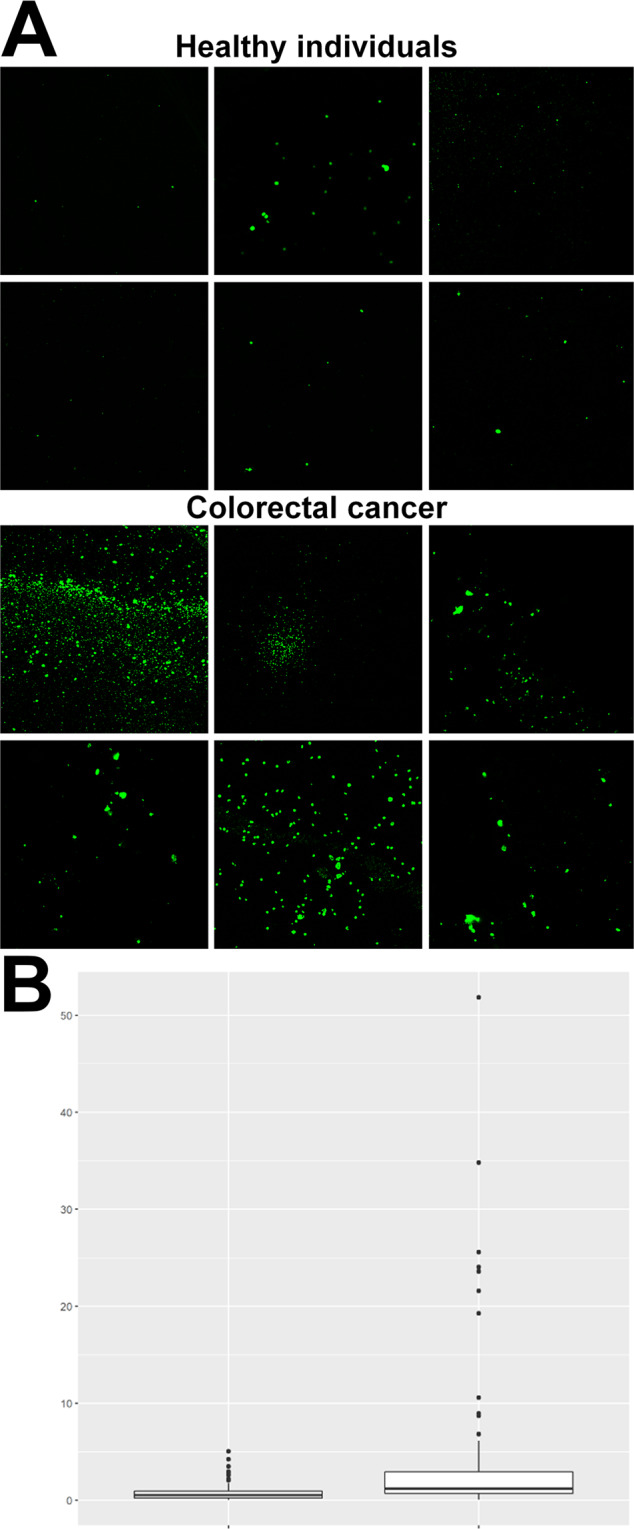
(**A**) Representative platelet poor plasma (PPP) smears with added anti-*E. coli* LPS antibody and secondary antibody, from healthy individuals and colorectal cancer (CRC) patients. Micrographs were enhanced for publication clarity, by adjusting the brightness and contrast of the images in Adobe Photoshop CS6. (**B**) Box and whisker plot of the normalised (to secondary antibody control signal) mean fluorescence intensities (MFIs) of healthy (n = 29) (left box) and CRC (n = 25) (right box) PPP smears, with added anti-*E. coli* LPS antibody and secondary antibody.

A direct fluorescence LPS antibody-based technique was employed to determine the circulating levels of LPS in CRC patients, compared to that of healthy individuals. This technique has been optimized previously^27^. Representative confocal micrographs of PPP smears with added anti-*E. coli* LPS antibody and secondary antibody, from healthy individuals and CRC patients, are shown in Fig. 7A. Table 4 shows the normalised MFIs of the confocal images of PPP smears. There is a significant increase (at Bonferroni corrected levels) in the fluorescence LPS signal in PPP from CRC patients, in comparison with healthy PPP (refer to Fig. 7B for a box and whisker plot of the normalised MFIs of the confocal micrographs of healthy and CRC PPP smears). The fluorescence LPS signal was identified as a parameter with statistical significance by logistic regression modelling, with elevated circulating LPS levels being predictive of an increased chance of CRC (refer to Table 4).

**Table 4:** Normalised mean fluorescence intensities (MFIs) of confocal images of platelet poor plasma (PPP) smears with added anti-*E. coli* LPS antibody and secondary antibody, and MFIs (units are unscaled pixel intensity (0 to 255)) of confocal images of PPP clots (stained with Amytracker 630 and unstained).

	Healthy individuals	CRC patients	Unadjusted OR (99.8% CI)	Adjusted OR (99.8% CI)
**Anti-*E. coli*** **LPS antibody**	0.52 [0.25–0.95]	1.22 [0.71–2.93]	**2.2764 (1.5094–3.808)**	**2.1253 (1.372–3.6713)**
**Amytracker 630**	2.65 [2.16–2.97]	3.01 [2.58–4.02]	**2.6934 (1.3689–6.7481)**	**2.5903 (1.3436–6.4636)**
**Unstained**	1.55 [1.47–1.65]	1.51 [1.4–1.64]	1.4756 (0.13654–20.532)	1.7687 (0.13305–30.73)

Data expressed as median and [25−75% quartile range]. Significant differences are shown in bold for both models.

**Figure 8:**
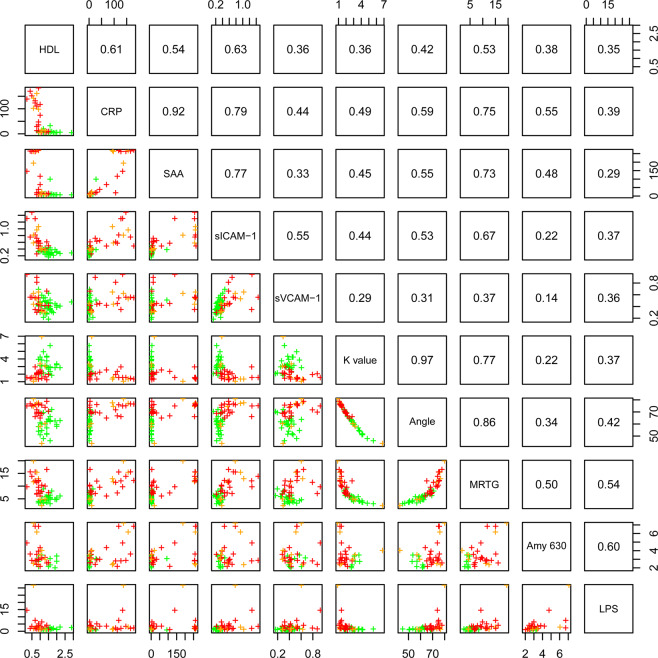
Lattice of cross-plots of selected biomarkers for colorectal cancer (CRC) across a wide range of groups, where the upper diagonal shows correlation coefficients (green = control population; orange = stage 2 CRC patients; red = stage 3 and stage 4 CRC patients).

Given the significantly elevated levels of LPS present in CRC PPP over healthy PPP, the correlation between LPS and other markers investigated in this paper, was calculated. Refer to Fig. 8 for a lattice of parameter cross-plots, where the upper diagonal shows the correlation coefficients and the lower diagonal shows pairwise scatter plots coloured by disease stage (green represents the control population, orange represents stage 2 CRC patients, and red represents stage 3 and 4 CRC patients). Linear regression between biomarkers (across a wide range of groups) and the fluorescence LPS signal firstly demonstrated that a significant correlation exists between LPS and TEG WB parameters (as shown in the cross-plot). The adjusted R^2^ values of 0.28 for MRTG and 0.16 for Angle are indicative of a positive relationship between a pathological coagulation system (hypercoagulability) and the presence of circulating LPS. CRP also correlates to LPS presence (R^2^ value of 0.15). Moreover, the pro-thrombotic mediators (or vascular damage markers) sICAM-1 and sVCAM-1 (R^2^ values of 0.12 and 0.11, respectively) also correlate with LPS levels, although not as strongly as the three TEG WB parameters and CRP.

### Aberrant blood clots and misfolded fibrin(ogen)

**Figure 9:**
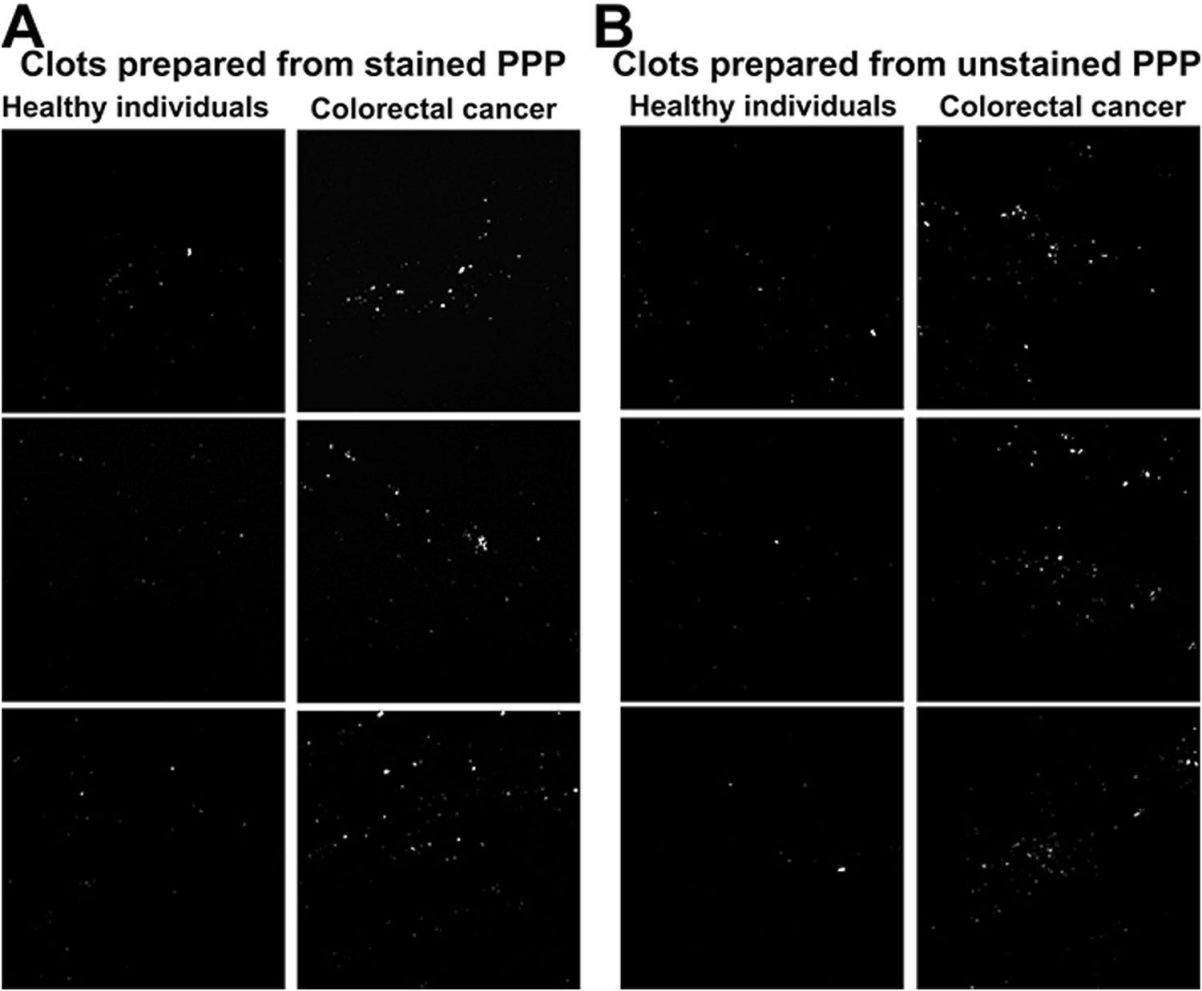
(**A**) Representative confocal micrographs of clots prepared from platelet poor plasma (PPP) stained with the amyloid-selective Amytracker 630 marker, showing the fluorescence (anomalous) amyloid signal of healthy individuals and colorectal cancer (CRC) patients. (**B**) Representative confocal micrographs of clots prepared from unstained PPP, showing the autofluorescence signal of healthy individuals and CRC patients. Micrographs were enhanced for publication clarity, by converting images to grayscale, followed by adjusting the brightness and contrast of the images in Adobe Photoshop CS6.

**Figure 10:**
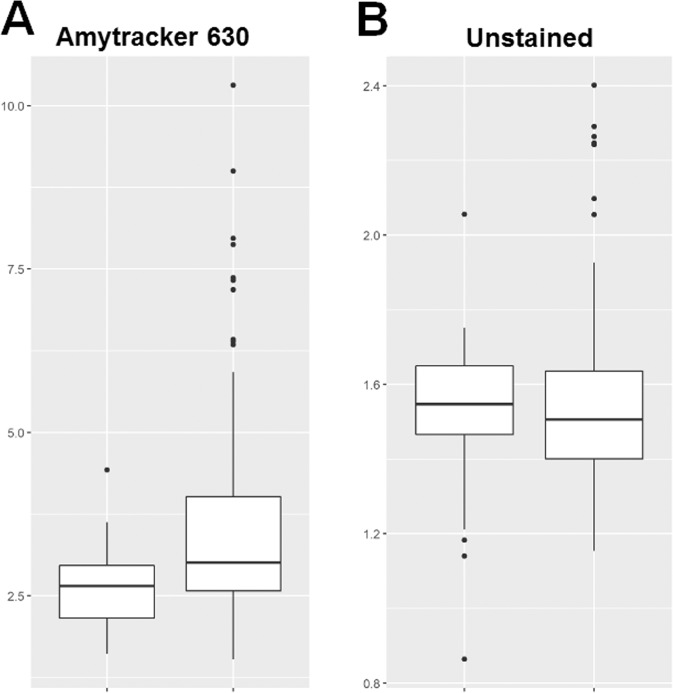
Box and whisker plots showing the distribution of the mean fluorescence intensities (MFIs) of (**A**) stained healthy (n = 10) (left box) and colorectal cancer (CRC) (n = 26) (right box) platelet poor plasma (PPP) clots and (**B**) unstained healthy (n = 10) (left box) and CRC (n = 26) (right box) PPP clots.

The presence of very low concentrations of LPS induces a change in the nature or structure of fibrin(ogen) by causing fibrin(ogen) plasma proteins to clot into an amyloid form^31,32^. Fibrinogen mainly consists of α-helices, but when amyloidogenesis occurs, fibrinogen undergoes a secondary structural change during polymerisation to fibrin^32^. PPP was incubated with the luminescent conjugated oligothiophene dye Amytracker 630, followed by the addition of thrombin to create a clot. Aberrant blood clots (anomalous clot formation) can be characterized by the binding of amyloid-selective stains, including Amytracker 630. Therefore, differences in protein structure and nature, specifically the presence of amyloid structures in PPP clots (an indication of pathological clotting) of CRC patients were compared to that of healthy individuals. MFIs were used to reflect the fluorescence (anomalous) amyloid signal. Representative confocal micrographs of clots prepared from stained PPP of healthy individuals versus clots prepared from stained PPP of CRC patients are shown in Fig. 9A. The MFIs of the control and CRC clot images were compared in a box and whisker plot, showing that the fluorescence signal in stained PPP clots from CRC patients is increased (Fig. 10A). Logistic regression modelling identified Amytracker 630 signal as a statistically significant parameter, but with smaller effect size than the fluorescence LPS signal (also refer to Table 4).

Moreover, in order to account for the effect of autofluorescence (a validation step), unstained PPP clots of healthy individuals were compared to unstained PPP clots of CRC patients. There is no significant difference in the autofluorescence signal (without the use of any fluorescent stain) of PPP clots from CRC patients, compared to PPP clots from healthy individuals (refer to Table 4 and to Fig. 10B for a box and whisker plot). This suggests that the fluorescence changes observed in the stained samples are not confounded by the presence of autofluorescence (intrinsic fluorescence) of these clots. Representative confocal micrographs of clots prepared from unstained PPP of healthy individuals versus clots prepared from unstained PPP of CRC patients are shown in Fig. 9B. Interestingly, the lattice of parameter cross-plots (Fig. 8) indicates that Amytracker 630 signal strongly correlates to the fluorescence LPS signal (adjusted R^2^ value of 0.36).

## Discussion

**Figure 11:**
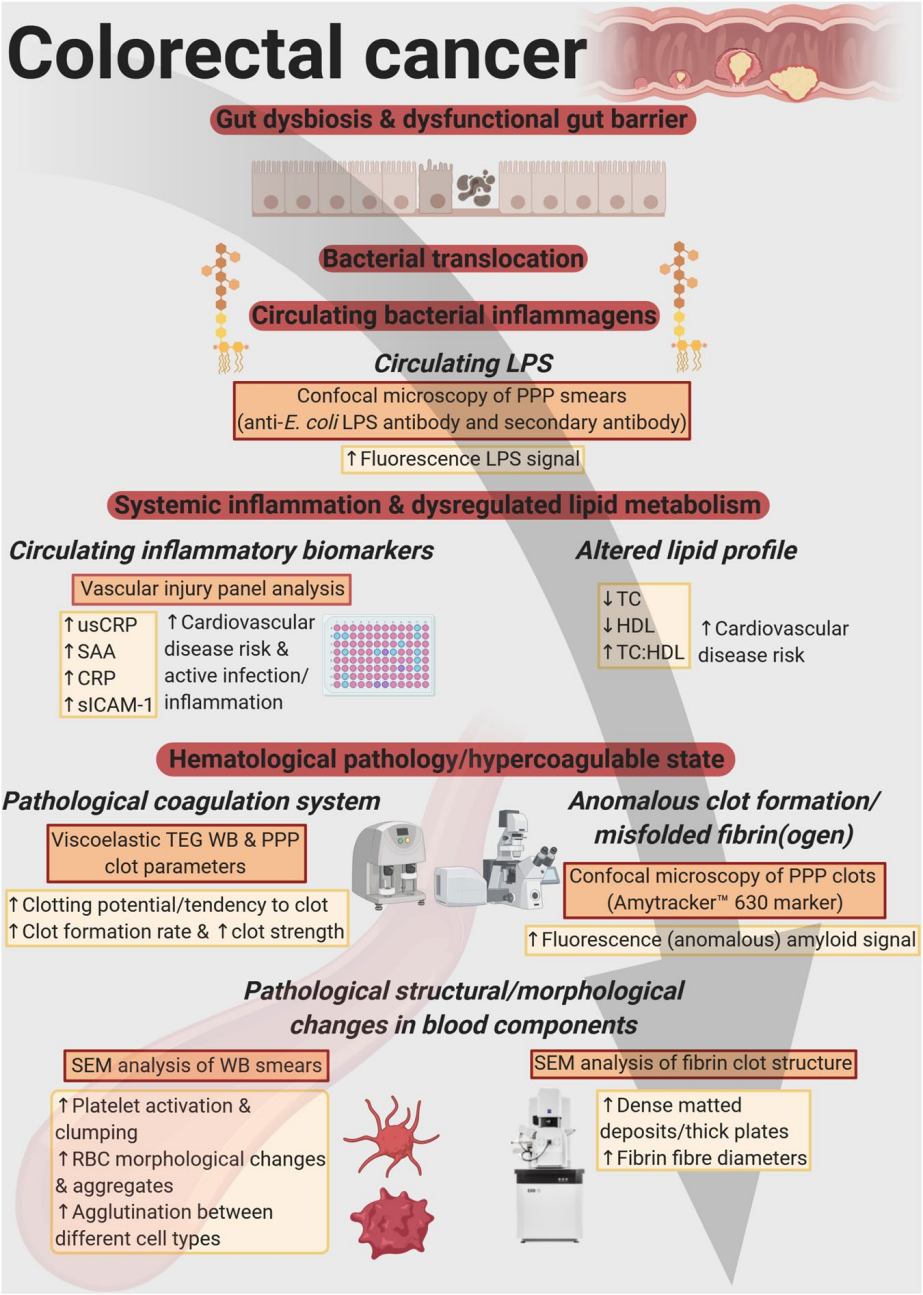
Summary of the results of the paper, highlighting circulating lipopolysaccharide (LPS) as one of the main factors promoting systemic inflammation and hematological dysfunction in colorectal cancer (CRC) patients. Figure made in ©BioRender - https://biorender.com/.

Our results are diagrammatically represented in Fig. 11 and the following paragraphs describe the relevant findings. We analysed and compared CRC patient blood samples to that of healthy controls in order to identify (discover) biomarkers/parameters that correlate to CRC status (to determine the strength of associations between biomarkers and CRC, and investigate their correlation to CRC stage/progression). This research aim could be achieved by employing logistic and ordinal regression, using healthy control and CRC data. It would fall outside the scope of this specific study to include blood samples from individuals with colorectal polyps as additional control samples, because it would not have had a significant impact on our approach in addressing our original research aim. Furthermore, we did not compare CRC cases to (chronic) inflammation cases, because our research aim could be achieved by comparing data of only CRC cases to healthy controls. Patients with Crohn’s disease or ulcerative colitis are at risk of developing colitis-associated cancer (CAC)^49^. This is a classical inflammation-driven or so-called “inflammation-induced” CRC^49^. Therefore, even though chronic inflammation acts as a preceding event that can lead to CRC, inclusion of such cases would not have added to achieving our original research aim.

We showed that the fluorescence *E. coli* LPS signal is significantly higher in PPP from CRC patients, compared to PPP from healthy individuals. Moreover, we found that there is a relationship or correlation between the presence of circulating LPS and CRP levels. This suggests that LPS is, to some degree, associated with the pro-inflammatory status observed in these patients. Infection, inflammation, and tissue injury lead to the production of CRP^50^, a widely used (non-specific) inflammatory marker^26^. SAA levels in the blood are also elevated in cases of trauma, infection, and inflammation^51^, acting as modulator and clinical marker of active inflammation^14,51^. Importantly, SAA can be upregulated up to 1000-fold in response to neoplasia^51^, and predicts a poor prognosis^52^. It has been reported that SAA, in comparison with other acute-phase proteins such as CRP, may serve as a more reliable, specific, and sensitive inflammatory biomarker^38,53^, also of disease activity in colorectal carcinoma^54^. The combination of elevated CRP and SAA levels, as seen in our CRC population, may be indicative of an increased risk of CRC^38^. Ordinal regression modelling also indicated that CRP and SAA levels have strong associations with CRC stage/progression. Importantly, as shown in the parameter cross-plot (Fig. 8), there exists a strong correlation between these two parameters.

It is important to note here that, in addition to acute-phase proteins, elevations in pro-inflammatory cytokines (such as interleukin-12 (IL-12) and interferon-γ (IFN-γ)) and cell populations are also representative markers of inflammation. It is known that the overexpression of T helper 17 (Th17) cells is implicated in tumorigenesis and associated with a poor prognosis^55^, whereas elevated expression of the Th1 cluster is associated with a better prognosis in CRC^56^. Furthermore, M1 macrophages, which are tumour associated macrophages (TAMs) with tumour preventing properties, have been shown to be associated with improved survival in CRC^57^.

SAA can activate the transcription factor NF-κB^58^, which plays important roles in regulating thrombosis^59^. SAA induces the expression of pro-thrombotic mediators, including sICAM-1 and sVCAM-1^26^. Ordinal regression modelling indicated that sICAM-1 levels correlate to the stage of CRC. It has been reported that sICAM-1 is associated with cancer progression, with sICAM-1 levels being indicative of the prognosis of CRC patients^60^. Furthermore, the significantly elevated ultrasensitive CRP levels in CRC patients are indicative of an increased risk for cardiovascular events and peripheral vascular disease^61^. Chronic inflammatory diseases, including CRC, are associated with an increased risk for atherosclerosis and ultimately cardiovascular disease^45,62^.

Long-term inflammation acts as an important hallmark of CRC^63^, and its role in tumour initiation, promotion, and progression is well-established^64^. Chronic systemic inflammation therefore forms a major part of CRC tumorigenesis^65–67^. Moreover, cross-talk exists between lipid metabolism and chronic inflammation. Dyslipidaemias, classically defined as decreased concentrations of circulating HDL cholesterol and elevated concentrations of triglycerides (hypertriglyceridemia)^68^, go hand in hand with chronic low-grade inflammation and infections^69,70^. CRC patients demonstrated significantly decreased HDL cholesterol levels, but none of the models have identified triglyceride as a statistically significant parameter. A study by Zhang and co-workers found that the serum levels of total cholesterol and LDL cholesterol in CRC patients were significantly lower than in healthy individuals, but no statistically significant difference in the serum levels of triglycerides between the two groups^71^. We also found that serum total cholesterol levels are significantly decreased in CRC patients, which correlates to increased CRC risk^72^. In line with this, ordinal regression modelling illustrated that total cholesterol levels are predictive of the stage or level of CRC. Abnormal lipid levels correlate to CRC risk^68^, but the role of cholesterol in carcinogenesis remains conflicting^73^.

A pro-inflammatory profile and coagulopathies such as a hypercoagulable state (pathological clotting) are linked^29,46^. Systemic (and localized) inflammatory processes can activate the coagulation system, causing systemic hypercoagulability^17^. The analysis of the viscoelastic clot parameters of CRC WB and PPP, as measured by TEG, is an effective approach to assess (hyper)coagulability (the efficiency of coagulation). TEG results indicated that WB and PPP samples from CRC patients have an increased tendency to develop a larger and denser clot quicker versus WB and PPP samples from healthy controls. Importantly, a significant positive correlation exists between LPS and markers of an activated or pathological coagulation system (TEG WB clot parameters). This suggests that coagulation dysfunctions may predict the presence of LPS and vice versa.

SEM analysis of WB smears conveyed valuable insights in the assessment of (hyper)coagulability. Distinct patho-morphological changes in the ultrastructure of platelets and RBCs, together with the increased agglutination between different cells, may add to the increased hypercoagulability in CRC patients, depicted by WB TEG results. The atypical non-discoid shapes of RBCs from CRC patients suggest RBC dysfunction, which may potentially lead to anaemic conditions. Anaemic conditions can be classified in different ways, of which one is based on the morphology or size of RBCs^74^. Furthermore, an increased number of eryptotic RBCs are present in CRC WB, which may lead or contribute to the development of anaemia^75,76^. It has been reported that anaemia serves as a sign of CRC^77^, and that cancer-associated anaemia is considered as a prognostic factor of the survival of cancer patients^75^.

Fibrin clot structure was also assessed with SEM analysis. As mentioned, fibrin(ogen) is the main determinant in PPP hypercoagulability, and SEM analysis of CRC fibrin clots confirmed an altered fibrin network ultrastructure, compared to healthy fibrin clots. It is clear form Fig. 6 and Table 3 that the diameters of fibrin fibres in clots created from CRC PPP are increased, compared to those of control PPP clots. CRC patients have a much larger amount of thick fibre counts than the control group, with thinner fibres appearing seldom. The dense matted surfaces observed in CRC fibrin clots are thus formed by thicker fibers, appearing as uniform plates, hence resulting in a hypercoagulable state (as suggested by PPP TEG results). The abnormal viscoelastic clot parameters measured by WB and PPP TEG analysis, as well as SEM analysis of WB smears and fibrin clot structure, suggest that blood components react to and undergo certain pathological changes in the presence of a dysregulated inflammatory milieu, ultimately affecting clot formation, causing clot hypercoagulability or an increased clotting potential. Circulating inflammatory markers therefore have the potential to change and activate the coagulation system, thereby promoting hematological pathology. It is known that malignancy leads to hypercoagulability, which forms part of tumorigenesis and metastasis via promoting angiogenesis^25,66^. An intricate relationship (bidirectional association) therefore exists between pathological hemostatic system activation and cancer^23,66^.

The anomalous (amyloidogenic) clotting of blood has been implicated in forming part of the origin of hypercoagulability in a variety of chronic inflammatory diseases^31,78^. Circulating LPS can induce the formation of anomalous or misfolded circulating fibrin(ogen), characterized by amyloid formation in fibrin(ogen), with the addition of thrombin (when forming a clot)^31,32^. We showed that CRC PPP clots contain significantly greater (anomalous) amyloid-specific signal than control PPP clots, by using the amyloid-selective Amytracker 630 marker. It has also been reported that LPS is closely associated with dense matted (amyloid) fibrin(ogen) deposits, which highlights the important role that LPS may play in abnormal blood clotting or hypercoagulation^27^. The strong correlation between Amytracker 630 signal and LPS presence further motivates that LPS may play an important role in anomalous clot formation in CRC patients. Evidence is therefore presented that elevated levels of circulating LPS, dysregulated levels of circulating inflammatory markers, and a pro-coagulant state are associated with CRC. We suggest that the statistically significant parameters identified in this study can be used for CRC screening, because logistic and ordinal regression models indicated that these biomarkers are predictive of an increased chance of CRC and correlate to the stage of CRC. However, the relatively small sample size is a limiting factor of this study, which necessitates future research with larger sample populations. Additionally, different subgroups of patients should be identified in future studies. For example, CRC cases and (chronic) inflammation cases can be distinguished in order to investigate and compare characteristic signatures of inflammation between such cases.

## Conclusions

Elevated levels of circulating bacterial inflammagens (specifically LPS) upregulate a variety of inflammatory biomarkers, ultimately promoting systemic inflammation. Gut dysbiosis and ‘leaky gut’ conditions in CRC patients can thus contribute to their dysregulated inflammatory biomarker profile, which manifests in multiple pathological hematological changes. Importantly, systemic hypercoagulability and aberrant blood clotting are strongly associated with CRC status. The significant correlations observed between circulating LPS levels and TEG WB clot parameters, suggest that a link exists between a bacterial presence and hematological pathology. Because of the intricate relationship between a pathological coagulation system and chronic inflammation in the pathogenesis of CRC (influencing malignant growth), new therapeutic strategies targeting this bidirectional network should be explored^66^. The wider relevance of this paper lies in the identification of biomarkers of multiple groups (inflammatory markers, vascular damage markers, and circulating LPS), that are associated with CRC and can be used for screening of CRC. Blood-based inflammatory markers can be used in combination with conventional CRC screening approaches, and may serve as additional tools for early detection of CRC^1^. With the help of effective CRC screening with the aim of early detection, followed by early intervention, the incidence of CRC mortalities can be significantly reduced^1,79^.

## Data Availability

The datasets generated during and/or analysed during the current study are available in the Onedrive Blood Laboratory Repository, https://1drv.ms/u/s!AgoCOmY3bkKHieA1W7n-DHeqiJTcZw?e=UglJJ5.
